# Delivering High-Quality Family Planning Services in Crisis-Affected Settings II: Results

**DOI:** 10.9745/GHSP-D-14-00112

**Published:** 2015-03-02

**Authors:** Dora Ward Curry, Jesse Rattan, Shuyuan Huang, Elizabeth Noznesky

**Affiliations:** a CARE USA, Atlanta, GA, USA.

## Abstract

An estimated 43 million women of reproductive age experienced the effects of conflict in 2012. Already vulnerable from the insecurity of the emergency, women must also face the continuing risk of unwanted pregnancy but often are unable to obtain family planning services. The ongoing Supporting Access to Family Planning and Post-Abortion Care (SAFPAC) initiative, led by CARE, has provided contraceptives, including long-acting reversible contraceptives (LARCs), to refugees, internally displaced persons, and conflict-affected resident populations in Chad, the Democratic Republic of the Congo (DRC), Djibouti, Mali, and Pakistan. The project works through the Ministry of Health in 4 key areas: (1) competency-based training, (2) supply chain management, (3) systematic supervision, and (4) community mobilization to raise awareness and shift norms related to family planning. This article presents data on program results from July 2011 to December 2013 from the 5 countries. Project staff summarized monthly data from client registers using hard-copy forms and recorded the data electronically in Microsoft Excel for compilation and analysis. The initiative reached 52,616 new users of modern contraceptive methods across the 5 countries, ranging from 575 in Djibouti to 21,191 in Chad. LARCs have predominated overall, representing 61% of new modern method users. The percentage of new users choosing LARCs varied by country: 78% in the DRC, 72% in Chad, and 51% in Mali, but only 29% in Pakistan. In Djibouti, those methods were not offered in the country through SAFPAC during the period discussed here. In Chad, the DRC, and Mali, implants have been the most popular LARC method, while in Pakistan the IUD has been more popular. Use of IUDs, however, has comprised a larger share of the method mix over time in all 4 of these countries. These results to date suggest that it is feasible to work with the public sector in fragile, crisis-affected states to deliver a wide range of quality family planning services, to do so rapidly, and to see a dramatic increase in the percentage of users choosing long-acting reversible methods.

## Abstract (precis)

A family planning program in 5 crisis-affected settings reached more than 52,000 new contraceptive users in just 2.5 years. Long-acting reversible contraceptives (LARCs) made up 61% of the method mix, with implants predominating in most countries. IUD use also increased over time as the program intensified its efforts to improve provider skills and user awareness. These findings demonstrate the strong popularity of LARCs and the feasibility of providing them in fragile settings even though they require more training and infrastructure support than short-acting methods.

## BACKGROUND

In 2012, an estimated 61 million people needed humanitarian assistance for food and non-food items, water, sanitation, and health services in response to conflict and natural disasters.^1^ Approximately 43 million women of reproductive age experienced the effects of conflict, and more than 15 million women of reproductive age were in acute need of humanitarian aid.^1^

Women in crisis situations are already vulnerable from the insecurity and disruption of the emergency, and they also must face the continuing risk of unwanted pregnancy. Furthermore, women are at increased risk of sexual violence and its consequences (physical and emotional trauma, unwanted pregnancy, unsafe abortion) due to the insecurity and increased violence that often accompany humanitarian emergencies. Many women would choose not to be pregnant in these situations, but they are often unable to obtain family planning services.^2^

Many women in crisis situations would choose not to be pregnant, but they are often unable to obtain family planning services.

Women in crisis-affected settings are also at greater risk of pregnancy-related illness and death due to interruptions or restricted access to pregnancy and delivery-related services. Worldwide, 9 of 10 countries with the highest maternal mortality ratios are affected by conflict.^3^ These high maternal mortality ratios are related not only to poor emergency obstetric care but also to high unmet need for family planning.

CARE's ongoing Supporting Access to Family Planning and Post-Abortion Care in Emergencies (SAFPAC) initiative operates in Chad, the Democratic Republic of the Congo (DRC), Djibouti, Mali, and Pakistan—5 crisis-affected countries that face persistent challenges including recurrent natural disasters, chronic conflict, and significant populations of internally displaced persons (IDPs) or refugees. SAFPAC supports government health systems at the provincial level and below to apply family planning best practices established in more stable development settings to crisis-affected settings for refugees, host country communities, IDPs, and other crisis-affected residents.

The countries and geographic areas in which SAFPAC operates face a diverse range of challenges. Eastern DRC suffers protracted conflict, insecurity, and chronic displacement of people. Southern Chad is managing an influx of refugees from the Central African Republic. Major, recurrent flooding (and displacement) affect South Punjab, Pakistan. North-central Mali hosts IDPs from the far North. Djibouti hosts Somalian refugees in stable, long-running camps.

Although very different from one another, these countries share some important similarities. First, protracted conflict and/or natural disasters have caused large population displacement and have eroded the ability of the state to provide basic services, which in most cases are already weak. Second, unmet need for modern contraception is high and increases in contraceptive prevalence have been slow, resulting in their selection as Family Planning 2020 (FP2020) focus countries.^4^ Contraceptive prevalence nationally ranges from less than 10% in Chad, the DRC, and Mali to 18% in Djibouti and 22% in Pakistan (Table 1). Third, their governments have signaled at least some commitment to increasing access to family planning services through various national policies and programs.

**Table 1.** SAFPAC Project Settings

**Country**	**Countrywide CPR for Modern Methods^a^**	**No. of SAFPAC Facilities**	**Estimated No. of WRA in Catchment Area^b^**
Chad	5%	21	405,984
DRC	8%	21	123,218
Djibouti	18%^c^	2	5,040
Mali	8%	8	14,210
Pakistan	22%	27	149,601
**Total**	**NA**	**79**	**698,053**

Abbreviations: CPR, contraceptive prevalence rate; DRC, Democratic Republic of the Congo; NA, not applicable; SAFPAC, Supporting Access to Family Planning and Post-Abortion Care; WRA, women of reproductive age.

a Source of countrywide CPR data: Pakistan 2012–13 Demographic and Health Survey (DHS),^5^ DRC 2013–14 DHS,^6^ Mali 2012–13 DHS,^7^ UNFPA 2011 State of the World's Midwifery (for Djibouti),^8^ and Chad 2010 Multiple Indicator Cluster Survey.^9^

b These population figures may be inaccurate estimates for 2 reasons: (1) The catchment populations of regional and districts hospitals often contain the catchment populations of health centers that SAFPAC supports, leading to double-counting. (2) There were changes in the number and type of facilities that SAFPAC supported between 2012 and 2013, when the second phase of programming began, thus leading to changes in the actual population covered.

c The CPR for modern methods was documented to be much lower in the Djibouti camps where SAFPAC works (5.1%).^10^

SAFPAC's implementation strategy focuses on 4 intervention areas, which have been shown to increase access to and use of family planning services in any setting^11^^–^^15^: (1) providing competency-based training with follow-up assessment and coaching, (2) conducting facility supervision on a regular basis in partnership with local government health officials, (3) ensuring a continuous supply of contraceptives and medical supplies, and (4) mobilizing communities to raise awareness about family planning and to shift norms that block women's access to services.

CARE teams collaborate closely with provincial, district, and health facility management, service delivery staff, and community partners, on both overall design and day-to-day implementation of the program. SAFPAC supports basic first-level health centers, health centers providing basic delivery, and district and some regional/provincial hospitals, and works with a range of mid- and higher-level providers, including nurses, medical technicians, Lady Health Visitors (community health workers), and doctors. The initiative supports the entire range of contraceptive methods sanctioned by the government in each country and also supports postabortion care (PAC), which includes family planning services. The number of women of reproductive age in the project catchment areas ranges from 5,040 in Djibouti to 405,984 in Chad (Table 1).

For more details about the interventions and lessons learned during implementation, see the companion article in *Global Health: Science and Practice*.^16^ In this article, we show the effects of the initiative's interventions on modern method use, using project monitoring data over the first 2.5 years of the project.

## METHODS

We analyzed project service delivery data from July 2011 to December 2013 from all 5 countries. The program collected these data for the purpose of continuous quality improvement, not for the purpose of conducting systematic research in a strictly defined model.

We focused data collection on the 4 contraceptive methods that are commonly offered at all health facilities—oral contraceptive pills, injectable contraceptives, implants, and intrauterine devices (IUDs). We also analyzed use of permanent methods, but they are offered in only a few facilities that we support. Health workers provide condoms and discuss their advantages and disadvantages in counseling; however, we do not track new condom users at the program, country, or global level to avoid double-counting those using them in combination with other methods and because of their relatively low effectiveness in reducing unwanted pregnancy during typical use. We also do not track use of emergency contraceptives at the program's national or global level because they are not an approved method in some countries.

The indicators reviewed in this article are the number and percentage of new users by contraceptive method, the percentage of new family planning users who adopted long-acting, reversible contraceptives (LARCs), and the number and percentage of PAC clients who adopted contraceptive methods.

### Data Collection and Management

**Figure 1.**
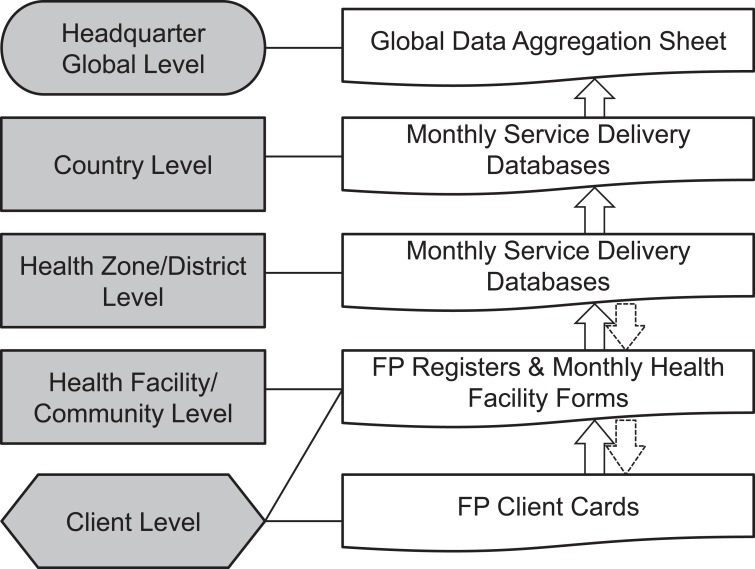
SAFPAC Assessment Tools and Data Flow Chart Abbreviations: FP, family planning; SAFPAC, Supporting Access to Family Planning and Post-Abortion Care.

The project's patient recordkeeping and data collection system has been integrated into the government's health information system. Health facilities assign each client an individual client card, which serves as a record of the client's history and previous visits. In addition, family planning registers record details of each visit by client encounter. Each month, project staff or providers summarize the data from family planning registers from each facility and collect them in hard-copy forms designed specifically for the project. Then project staff record the data electronically in Microsoft Excel and send them to the SAFPAC country-level management team. At the country level, the management team analyzes the date to explore trends that could inform decision making, and then the team sends the data to headquarters for compilation and further analysis (Figure 1).

Supervisors conduct data quality checks regularly during monthly supervisory visits by comparing summary monthly reports, patient records, and visit registers. As the project has progressed, the supervisors have begun reporting the findings of their monthly supervisory checklists to the district, national, and global level as well. (See companion article in *Global Health: Science and Practice* for more information about our supervisory approach.^16^)

### Data Analysis and Use

In the health facilities, providers use wall charts to track service delivery progress. Project and government staff meet regularly at both facility and district levels to assess service delivery patterns and unexpected changes and to adjust programming as necessary. These teams also hold joint meetings with community leaders monthly or quarterly to discuss trends, develop specific action plans, and document next steps in handwritten supervisory logs. At the health zone/district and country levels, program staff use an Excel datasheet with built-in formulas and automatically generated graphics for data analysis. A globally aggregated datasheet compiles data and generates pivot tables and pivot charts for descriptive analysis.

## RESULTS

### New Modern Method Users

The first 2.5 years of the project have been characterized by high and sustained demand for modern methods of family planning in all 5 countries. In total, the initiative has reached 52,616 new users of modern methods in Chad, Djibouti, the DRC, Mali, and Pakistan (Table 2). The number of new modern method users has varied by country, from 575 in Djibouti to 21,191 in Chad.

**Table 2.** Number of New Modern Method Users and Distribution of LARCs vs. Other Modern Methods Among SAFPAC Initiative Facilities, by Country (July 2011–December 2013)

**Country**	**Any Modern Method^a^**	**LARCs**		**Other Modern Methods**	**% of LARCs Among All Modern Methods**
		**Implants**	**IUDs**		
Chad	21,191	13,201	2,189	5,841	72%
DRC	14,869	9,132	2,289	3,310	78%
Djibouti	575	6	0	569	1%
Mali	3,093	1,317	270	1,506	51%
Pakistan	12,888	298	3,432	9,158	29%
**Total**	**52,616**	**23,852**	**8,380**	**20,384**	**61%**

Abbreviations: DRC, Democratic Republic of the Congo; IUDs, intrauterine devices; LARCs, long-acting reversible contraceptives; SAFPAC, Supporting Access to Family Planning and Post-Abortion Care.

a Modern methods consisted of implants, injectables, IUDs, oral contraceptive pills, tubal ligation, and vasectomy.

**Figure 2.**
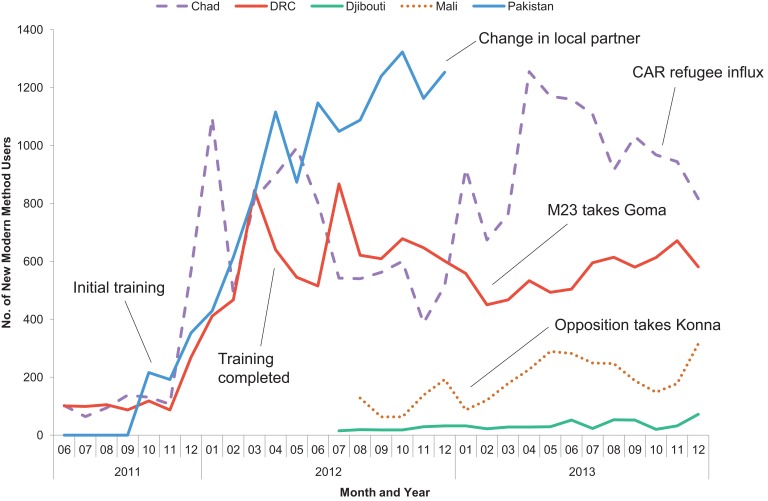
Number of New Modern Method Users Among SAFPAC Facilities, by Country and Month (July 2011–December 2013) Abbreviations: CAR, Central African Republic; DRC, Democratic Republic of the Congo; M23, March 23 Movement, also known as the Congolese Revolutionary Army; SAFPAC, Supporting Access to Family Planning and Post-Abortion Care.

An increase in new users was apparent immediately as trained providers and commodities became available. The first cohorts of providers completed their training in November 2011. Beginning in December 2011, a sharp increase in new users occurred in Chad, the DRC, and Pakistan (the 3 SAFPAC country programs active at the time). The upward trend continued through April 2012 as the final cohorts of providers received training (Figure 2). Subsequently, the 3 initial countries all experienced spikes and dips in monthly numbers of new users, many of which were correlated with changes in the active conflict (in the DRC) or recurring natural disasters (in Pakistan). Another major factor in periodic decreasing user trends was cultivation seasons, as SAFPAC covers primarily rural farming populations.

The project attracted new family planning users as trained providers and contraceptive commodities became available.

### Method Mix

Use of LARCs has predominated, representing 61% of total new users of modern methods across the 5 countries (Table 2). This overall average masks differences between countries. For example, in the DRC and Chad, 78% and 72% of new users, respectively, have chosen LARCs. In Mali, 51% of new users have chosen LARCs while in Pakistan, the proportion has been 29%. In Djibouti, providers received training in implant and IUD insertion at the end of 2013, so those methods were not offered in the country through SAFPAC during the period discussed here.

**Figure 3.**
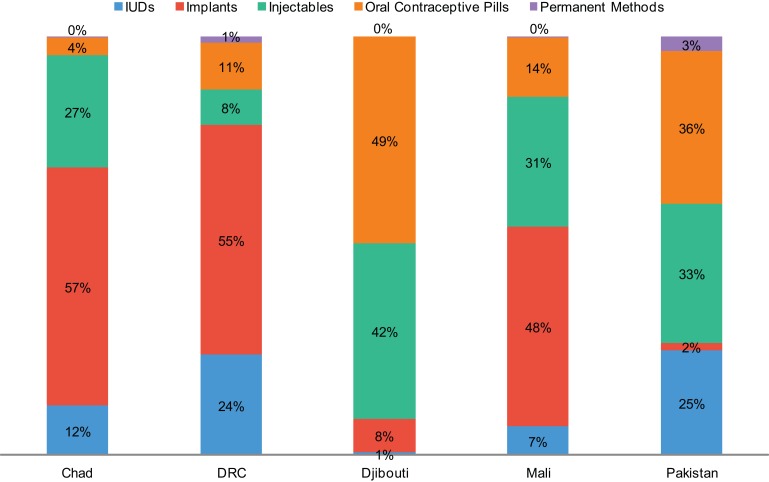
Method Mix Among New Modern Method Users in SAFPAC Facilities, by Country (July 2011–December 2013) Abbreviations: DRC, Democratic Republic of the Congo; IUDs, intrauterine devices; SAFPAC, Supporting Access to Family Planning and Post-Abortion Care.

Between the 2 LARC methods, use of implants has predominated in the African countries (excluding Djibouti) while IUD use has represented a small portion of LARC use (Figure 3), at 14% in Chad, 17% in Mali, and 21% in the DRC. Practically speaking, the popularity of implants has coincided with the introduction and availability of the method; before SAFPAC began its activities, implants were virtually unavailable in these settings except in private-sector clinics in larger urban areas. In Pakistan, on the other hand, IUDs have been the predominant LARC method, at 92% of total LARC use. This is due, in part, to difficulty in securing commodities and governmental approval for the use of implants.

**Figure 4.**
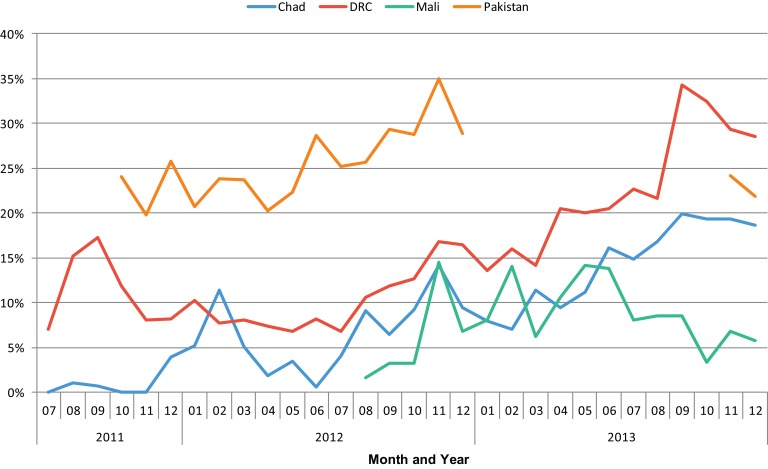
Share of IUDs of the Total Method Mix in SAFPAC Facilities, by Country and Month (July 2011–December 2013) Abbreviations: DRC, Democratic Republic of the Congo; IUDs, intrauterine devices; SAFPAC, Supporting Access to Family Planning and Post-Abortion Care.

In all 4 countries that offer IUDs, use of IUDs has comprised a larger share of the method mix over time. For example, IUDs as a percentage of total new users has shifted in the DRC from 11.3% in the first 6 months of project activities to 28.2% in the most recent 6 months, and from 1% to 18% in Chad. In Mali, the share of IUDs has increased only slightly, from 6.2% to 6.8%. In Pakistan, IUDs represented a higher percentage of total new users initially than in other countries, at 23.0%, but the share has still increased to 28.8% (Figure 4).

### Contraceptive Use Among PAC Clients

On average, over the project period, 28% of PAC clients in Djibouti have adopted a contraceptive method, compared with 57% and 59% in Chad and Pakistan, respectively, 65% in the DRC, and 77% in Mali.

## DISCUSSION

Using project monitoring data, the SAFPAC initiative has demonstrated the feasibility of providing high-quality family planning services, including long-acting methods, in fragile settings and at a rapid pace. Over the initial 2.5-year period, the project reached 52,616 new modern method users among a catchment population of about 698,000 women of reproductive age in 5 crisis-affected country settings across Africa and Asia, exceeding the project's target of 36,002 new users over the corresponding project period.

Of particular note is the dramatic increase in use of LARCs as a percentage of the overall method mix, in particular in the sub-Saharan African countries of Chad, Mali, and the DRC. LARCs are increasingly representing a greater portion of the method mix generally throughout sub-Saharan Africa. However, debate continues about the feasibility of providing LARCs in crisis-affected settings. The requirements both for provider training and for basic infrastructure required to insert IUDs and implants are significantly greater than those to dispense pills or administer injectable contraceptives. In SAFPAC-supported facilities, LARCs comprised 61% of the method mix, demonstrating not only that it is feasible to provide these methods in such settings but also that women in crisis-affected settings want and will use LARCs when the methods are available and they know about them, supporting earlier findings.^17^

Providing long-acting methods in fragile setting is feasible, even though they require more training and infrastructure support than short-acting methods.

Sullivan et al. note that while there is generally no ideal method mix, there may be reason for concern when 1 or 2 methods predominate in a country.^18^ Use of implants has predominated in the areas supported by the SAFPAC initiative. Implants seem to appeal to both providers and clients. Providers at SAFPAC-supported clinics reported liking the ease and speed of implant insertion. Clients reported liking that the insertion procedure does not require disrobing (as with IUDs) and ease of using the method (no need to return for refill/reinjection). In addition, our conversations with communities, clients, and providers suggest that other major factors behind women choosing implants include availability of the method free of charge in SAFPAC-supported facilities, in contrast to private channels where the method is available but very expensive, and word-of-mouth through rural social networks.

Still, the project team wanted to ensure that the higher use of implants over IUDs was not the result of misinformation or bias against IUDs. Accordingly, the SAFPAC team conducted additional provider training to offer unbiased counseling, refresher training to increase their confidence in IUD insertion and removal, and tightened and improved messaging related to the advantages of IUDs.

Project efforts to increase access to IUDs appear to be correlated with the upward trend of increasing IUD use in Chad, the DRC, and Pakistan. In DRC facilities, in particular, the share of IUDs increased to more than 20% of the total method mix compared with an overall sub-Saharan African average of 3%.^19^ We attribute this increase to 2 main factors: (1) Project supervisors began to clearly reinforce to providers the potential advantages of IUDs and the importance of presenting all options to women using our adapted DMT/BCS+ approach (even if they had heard of a different method before arriving for services), and (2) As more women began to try IUDs, influential satisfied clients, as well as male and female community leaders, shared positive experiences through their social networks.

Use of IUDs increased over time as the project intensified its efforts to improve provider training and potential clients' awareness.

In terms of family planning adoption among PAC clients, the project aims to meet a standard of 80% of PAC clients adopted a contraceptive method. The average rate of family planning acceptance among PAC clients, however, ranges from 3.2% in areas with no supportive interventions to anywhere from 45% to over 90% after implementing interventions.^20^ In Mali, 77% of PAC clients at SAFPAC facilities have adopted family planning, which is near the global target of 80%. Although our facilities in the other 4 countries have not yet reached this target, we believe they are on the path to doing so, with current levels between 28% (in Djibouti) to 65% (in the DRC).

### Strengths and Limitations

Our ability to assess true coverage of family planning services among the general population in the regions we cover, and to show causal relationships or make generalizable conclusions, is limited because the findings presented in this article come from routine monitoring data, not population-based surveys of randomly sampled, representative groups. While we have some information from client records on the characteristics of users, we cannot conclude with confidence that this group of users is fundamentally the same as the general population of women of reproductive age in terms of access, age, income level, and other demographic characteristics. Instead of diverting resources to such costly population-based surveys, the project has instead focused on collecting high-quality routine monitoring data and making maximum possible use of the data to continually improve the program.

We also face challenges in our ability to attribute the increase in new users of family planning to our efforts because we lack a comparison group among surrounding populations. It was not within the scope of the program to create such a comparison group. We are currently seeking ways to design a low-cost approach to collect household information to estimate contraceptive prevalence and unmet need and to determine whether women are unable to access services due to geographic distance, social or economic marginalization, or other unknown reasons. However, the notably higher numbers of clients per month and the rapid increase in contraceptive uptake shortly after trained providers and commodities became available do suggest that SAFPAC is meeting preexisting unmet need through high-quality service delivery. Furthermore, the sustained uptake in subsequent months suggests that CARE's community engagement activities are effective in accessing latent demand even after initial pent-up unmet need had been satisfied.

## CONCLUSION

These results suggest that it is feasible to work with the public sector in fragile crisis-affected states across diverse settings to offer a wide range of family planning services and methods. Moreover, the project demonstrated that it is feasible to do so in a short time period (about 2 years), resulting in rapid uptake of family planning overall, as well as a dramatic increase in the percentage of users voluntarily choosing highly effective long-acting reversible methods. This can be done using a set of interventions, drawn from best practices in development settings, that tackle typical service delivery challenges, increase awareness of a range of different methods, and begin to challenge norms that restrict women's access to family planning. Marshalling resources to reach women in a wide range of crisis-affected settings worldwide could make a meaningful contribution to progress on Millennium Development Goal No. 5 to improve maternal health.

## Notes

**Peer Reviewed**

**First published online:** 2015 Feb 4

## References

[1] Development Initiatives. Global humanitarian assistance (GHA) report 2012. Bristol: Development Initiatives; 2012 Available from: http://www.globalhumanitarianassistance.org/report/gha-report-2012

[2] McGinn T Reproductive health of war-affected populations: what do we know? Int Fam Plan Perspect. 2000;26(4): 174–180 10.2307/2648255

[3] World Health Organization (WHO); United Nations Children's Fund (UNICEF); United Nations Population Fund (UNFPA); World Bank. Trends in maternal mortality: 1990 to 2008. Geneva: WHO; 2010 Available from: http://www.who.int/reproductivehealth/publications/monitoring/9789241500265/en/

[4] Family Planning 2020 (FP2020). FP2020 partnership in action 2012–2013. Washington (DC): FP2020; 2013 Available from: http://advancefamilyplanning.org/sites/default/files/resources/FP2020_PartnershipInAction_2012–2013_lores.pdf

[5] National Institute of Population Studies (NIPS) [Pakistan]; ICF International. Pakistan demographic and health survey 2012–13. Islamabad (Pakistan): NIPS; 2012 Co-published by ICF International. Available from http://dhsprogram.com/publications/publication-FR290-DHS-Final-Reports.cfm

[6] Ministère du Plan et Suivi de la Mise en oeuvre de la Révolution de la Modernité (MPSMRM); Ministère de la Santé Publique (MSP); ICF International. Enquête démographique et de santé en République Démocratique du Congo 2013–2014. Rockville (MD): ICF International; 2014. Co-published by MPSMRM and MSP. Available from: http://dhsprogram.com/publications/publication-FR300-DHS-Final-Reports.cfm

[7] Cellule de Planification et de Statistique (CPS/SSDSPF); Institut National de la Statistique (INSTAT/MPATP); INFO-STAT; ICF International. Enquête démographique et de santé au Mali 2012–2013. Rockville (MD): ICF International; 2014. Copublished by CPS, INSTAT, and INFO-STAT. Available from: http://dhsprogram.com/publications/publication-FR286-DHSFinal-Reports.cfm

[8] United Nations Population Fund (UNFPA). The state of the world's midwifery 2011. New York: UNFPA; 2011 Available from: http://www.unfpa.org/sites/default/files/pub-pdf/en_SOWMR_Full.pdf

[9] United Nations Children's Fund (UNICEF). Résume exécutif. Enquête par grappes à indicateurs multiples: Tchad 2010. New York: UNICEF; 2011 Available from: http://www.childinfo.org/files/MICS4_Chad_2010_SummaryReport_Fr.pdf

[10] United Nations High Commissioner for Refugees (UNHCR); Women's Refugee Commission; Centers for Disease Control and Prevention. Baseline study: family planning among Somali refugees in Ali Addeh, Djibouti. New York: Women's Refugee Commission; 2011 Available from: http://www.unhcr.org/4ee6152b9.pdf

[11] Delker PV Basic skills education in business and industry: factor for success or failure. Washington (DC): United States Congress, Office of Technology Assessment; 1990.

[12] Jonas E Improving provider performance: an exploration of the literature. MotherCare Matters. 2000;9(2): 1–23 Available from: http://www.jsi.com/JSIInternet/Inc/Common/_download_pub.cfm?id = 10510&lid = 3

[13] Marquez L Kean L Making supervision supportive and sustainable: new approaches to old problems. Washington (DC): United States Agency for International Development; 2012 Available from: https://www.k4health.org/sites/default/files/maqpaperonsupervision.pdf

[14] Rowe AK de Savigny D Lanata CF Victora CG. How can we achieve and maintain high-quality performance of health workers in low-resource settings? Lancet. 2005;366(9490): 1026–1035. 10.1016/S0140-6736(05)67028-6. 16168785

[15] Rosato M Laverack G Grabman LH Tripathy P Nair N Mwansambo C. Community participation: lessons for maternal, newborn, and child health. Lancet. 2008;372(9642): 962–971. 10.1016/S0140-6736(08)61406-3. 18790319

[16] Curry DW Rattan J Nzau JJ Giri K Delivering high-quality family planning services in crisis-affected settings I: program implementation. Glob Health Sci Pract. 2015;3(1): 14–24 10.9745/GHSP-D-14-00164 PMC4356272 25745117

[17] Casey SE McNab SE Tanton C Odong J Testa AC Lee-Jones L. Availability of long-acting and permanent family-planning methods leads to increase in use in conflict-affected northern Uganda: evidence from cross-sectional baseline and endline cluster surveys. Glob Public Health. 2013;8(3): 284–297. 10.1080/17441692.2012.758302. 23305269 PMC3613974

[18] Sullivan TM Bertrand JT Rice J Shelton JD. Skewed contraceptive method mix: why it happens, why it matters. J Biosoc Sci. 2006;38(04): 501–521. 10.1017/S0021932005026647. 16762087

[19] May K Ngo TD Hovig D Expanding contraceptive choices for women: promising results for the IUD in sub-Saharan Africa. London: Marie Stopes International; 2011 Available from: https://mariestopes.org/sites/default/files/Expanding-contraceptive-choices-for-women-FINAL.pdf

[20] Tripney J Kwan I Bird KS. Postabortion family planning counseling and services for women in low-income countries: a systematic review. Contraception. 2013;87(1): 17–25. 10.1016/j.contraception.2012.07.014. 22974595

